# Establishment of a single-center-based early prognostic scoring system for Guillain-Barré syndrome

**DOI:** 10.1186/s12883-023-03143-4

**Published:** 2023-03-04

**Authors:** Xiaomeng Di, Jiawei Wang, Lei Li, Lei Liu

**Affiliations:** 1 Department of Neurology, Beijing Tongren Hospital, Capital Medical University, Beijing, China; 2 Beijing Institute of Ophthalmology, Beijing Tongren Eye Center, Beijing Tongren Hospital, Capital Medical University; Beijing Ophthalmology & Visual Sciences Key Laboratory, Beijing, China

**Keywords:** Guillain-Barré syndrome, Early prognostic scoring systems, GBS disability score, Pneumonia, Hypoalbuminemia, Hyponatremia

## Abstract

**Background:**

Previous studies have developed clinical prognostic models for Guillain-Barré syndrome including EGOS and mEGOS, they have good reliability and accuracy, but individual entries are poor. This study aims to establish a scoring system to predict the early prognosis, in order to provide additional treatment for patients with poor prognosis and shorten the length of hospital stay.

**Methods:**

We retrospectively analyzed risk factors affecting the short-term prognosis of Guillain-Barré syndrome, and developed a scoring system for early determination of disease prognosis. Sixty two patients were divided into two groups based on the Hughes GBS disability score at discharge. Groups were compared for differences in gender, age at onset, antecedent infection, cranial nerve involvement, pulmonary infection, mechanical ventilation support, hyponatremia, hypoproteinemia, impaired fasting glucose, and peripheral blood neutrophil-to-lymphocyte ratio. Statistically significant factors were included in a multivariate logistic regression analysis, and a scoring system to predict the short-term prognosis was established based on the regression coefficients. The receiver operating characteristic curve of this scoring system was plotted, and the area under the ROC curve was calculated to assess the accuracy of the prediction model.

**Results:**

Univariate analysis revealed that age at onset, antecedent infection, pneumonia, mechanical ventilation support, hypoalbuminemia, hyponatremia, impaired fasting glucose, and elevated peripheral blood neutrophil-to-lymphocyte ratio were risk factors for poor short-term prognosis. The above factors were included in the multivariate logistic regression analysis, and pneumonia, hypoalbuminemia, and hyponatremia could be used as independent predictors. The receiver operating characteristic curve was plotted with a calculated area under the ROC curve of 82.2% (95% CI 0.775—0.950, *P* < 0.0001). The best cut-off value for the model score was 2, with a sensitivity of 0.9091, a specificity of 0.7255, and a Youden index of 0.6346.

**Conclusion:**

Pneumonia, hyponatremia, and hypoalbuminemia were independent risk factors for poorer short-term prognosis in patients with Guillain-Barré syndrome. The short-term prognosis scoring system of Guillain-Barré syndrome we constructed using these variables had some predictive value, and the short-term prognosis with quantitative scores of 2 or more was worse.

**Supplementary Information:**

The online version contains supplementary material available at 10.1186/s12883-023-03143-4.

## Introduction

Guillain-Barré syndrome (GBS) is a common immune-mediated autoimmune disorder of the peripheral nervous system characterized by rapidly progressive symmetrical weakness of the extremities, sensory abnormalities, and hypotonic reflexes. Molecular mimicry, anti-ganglioside antibodies, and complement activation may be involved in the pathogenesis of GBS. The disease tends to have a monochronic, self-limiting course, with symptoms mostly peaking at 2 weeks of onset [1]. Globally, approximately 10,000 patients develop GBS each year, and 20–30% of these patients may progress to respiratory failure [2]. Therefore, early prediction of poor prognosis and timely intervention and treatment are crucial.

In this study, we retrospectively analyzed the general data, clinical characteristics, and serological findings of 62 patients with GBS to investigate the independent predictors of poor early GBS prognosis, and to establish an early prognosis scoring system to guide disease treatment.

## Methods

A retrospective design was used to study patients with confirmed acute GBS who were admitted to the Department of Neurology at the Beijing Tongren Hospital of Capital Medical University from 2007 to 2021. (1) Inclusion criteria: (i) met the diagnostic criteria of the 2019 Chinese Guillain-Barré Syndrome Diagnosis and Treatment Guidelines developed by the Chinese Medical Association [3]; (ii) first-onset admission. (2) Exclusion criteria: (i) patients with combined definite intracranial lesions; (ii) patients with chronic inflammatory demyelinating polyradiculoneuropathy (CIDP); (iii) patients who could not be excluded from peripheral neuropathy caused by other etiologies; (iv) patients with incomplete case data.

Measured characteristics included gender, age at onset, antecedent infection (whether diarrhea, upper respiratory tract infection, pulmonary infection, or other unexplained infection occurred within 4 weeks prior to onset), cranial nerve involvement (presence of ophthalmoplegia, facial palsy, dysarthria, dysphagia, weak neck, and shoulder rotation), presence of pulmonary infection (symptoms such as cough, sputum, and fever during the course of the disease, and confirmation with high-resolution computed tomography (HRCT) of the lungs). Mechanical ventilatory support, hyponatremia, hypoalbuminemia, impaired fasting glucose and the peripheral blood neutrophil-to-lymphocyte ratio (NLR) were analyzed as alternative influencing factors. Peripheral blood was collected from all patients within 24 h of admission. Plasma sodium < 135 mmol/L was considered as combined hyponatremia. Fasting plasma glucose (FPG) > 6.1 mmol/L was considered impaired fasting glucose. Plasma albumin < 35 g/L was defined as hypoalbuminemia. An elevated NLR was defined as an NLR value > 2.135.

The GBS disability score developed by Hughes (Hughes functional grading scale, HFGS) [4] et al. was used for assessment on the day of discharge: 0 represented a completely normal state; 1 represented mild signs or symptoms and ability to run; 2 represented the ability to walk ≥ 10 m alone but the inability to run; 3 represented the ability to walk 10 m in open space with assistance; 4 represented a bedridden or wheelchair bound state; 5 represented a requirement of assisted ventilatory support; and 6 referred to death. Those with GBS disability scores > 3 at discharge were considered to have a poor early prognosis and those with GBS scores ≤ 3 had better early prognoses.

SPSS 23.0 and MedCalc statistical software were used for the analysis. The χ2 test or Fisher's exact test was used to compare groups of count data. (1) Univariate analysis was used to derive risk factors for poorer early prognosis (HFGS score > 3) in patients with GBS. (2) Statistically significant (*P* < 0.05) influencing factors obtained from this analysis were then included in a multivariate logistic regression analysis, and regression coefficients were calculated. (3) The integer value closest to the regression coefficient was used as the influencing factor score value in order to establish an early prognostic scoring system. (4) The predictive value of the scoring system was evaluated by plotting the receiver operating curve (ROC) curve: the area under the ROC curve (AUC) was calculated, the appropriate cut-off value was selected, and the sensitivity, specificity, positive predictive value, and negative predictive value were calculated.

## Results

### General data

Of the 62 patients included, 37 were male and 25 were female. Patients were aged between 10–78 years, with a median age of (43.24 ± 15.76) years. The length of hospital stay is 6–51 days, with a median (IQR) length of 15.5 (13–22) days. The clinical and laboratory data are summarized in Table 1. Thirty-four (54.8%) of the 62 patients were aged > 40 years, 45 (72.6%) had antecedent infections, 59 (95.2%) had cranial nerve involvement, 7 (11.3%) had pulmonary infections, 16 (25.8%) had hyponatremia, 12 (19.4%) had hypoproteinemia, 11 (17.7%) had impaired fasting glucose, and 32 (51.6%) had NLR scores > 2.135. Among the 62 included patients, 51 cases (82.3%) had GBS disability scores ≤ 3 and 11 cases (17.7%) had GBS disability scores > 3. An additional file shows this in more detail (see Additional file 1).

**Table 1:** Clinical and laboratory data of participants

Characteristic	Overall (*n* = 62)
Age (y), mean (± SD)	43.24 ± 15.76
Sex (Male/Female)	37/25
Length of hospital stay (d), median (IQR)	15.5 (13–22)
Serum sodium, median (IQR)	138.05 (134.375–141)
Serum albumin, median (IQR)	39.25 (36–42.25)
FPG, median (IQR)	5.07 (4.625–5.775)
NLR, median (IQR)	2.135 (1.701–4.8853)

Continuous data were presented as the mean (± standard deviation [SD]) for normally distributed data and as median (interquartile range[IQR]) for nonnormally distributed data

*FPG* Fasting plasma glucose, *NLR* Neutrophil-to-lymphocyte ratio

### Univariate analysis

Univariate analysis demonstrated no statistically significant differences in gender and cranial nerve involvement between the two groups. In contrast, age at onset, prodromal infection, co-infection of the lungs, need for mechanical ventilation, hyponatremia, hypoalbuminemia, impaired fasting glucose, and NLR did show statistically significant differences (*P* < 0.05) between the two groups (Table 2).

### Logistic regression analysis and establishment of the clinical prediction model

**Table 2:** Univariate analysis of risk factors for poorer early prognosis

GBS disability score		0–3	4–6	*P* value
n		51	11	
Length of hospital stay, median (IQR)		15(13–19)	24(18–36)	
Gender	Male	29	8	0.5008
	Female	22	3	
Age	≤ 40	27	1	0.0087
	> 40	24	10	
Antecedent infection	Yes	39	4	0.0256
	No	12	7	
Cranial nerve involvement	Yes	48	11	1
	No	3	0	
Pulmonary infection	Yes	2	5	0.012
	No	49	6	
Mechanical ventilatory support	Yes	1	4	0.0027
	No	50	7	
Hyponatremia	Yes	9	7	0.0041
	No	42	4	
Hypoalbuminemia	Yes	6	6	0.0042
	No	45	5	
Impaired fasting glucose	Yes	6	5	0.0187
	No	45	6	
NLR	High	23	9	0.0271
	Low	28	2	

Continuous data were presented as the mean (± standard deviation [SD]) for normally distributed data and as median (interquartile range[IQR]) for non-normally distributed data

*NLR* Neutrophil-to-lymphocyte ratio

The statistically significant influencing factors that we determined in the previous step were included in a multivariate logistic regression analysis model, and the input method was applied to screen the independent predictors. Ultimately, we determined that three indicators (pulmonary infection, hypoalbuminemia, and hyponatremia) could be used as independent predictors of poor early prognosis in GBS patients (Table 3).

**Table 3:** Logistic regression analysis of independent predictors of poor early prognosis in GBS patients

Independent predictors	Regression coefficient	Standard error	*P* value	Odds ratio	95%CI
Hypoalbuminemia	1.76664	0.90094	0.0499	5.8511	1.0008–34.2087
Pulmonary infection	2.31544	1.06116	0.0291	10.1294	1.2656–81.0710
Hyponatremia	1.91157	0.86507	0.0271	6.7637	1.2411–36.8596

GBS Guillain-Barré syndrome, *CI* Confidence interval

**Fig. 1:**
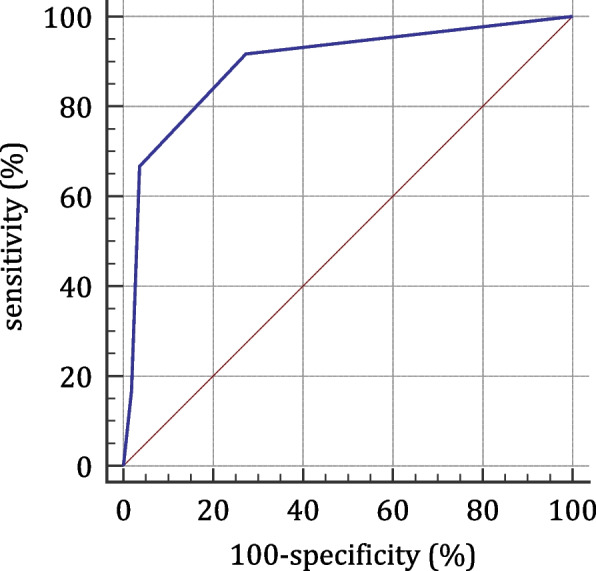
ROC curve showed the AUC of the PAN scoring system was 82.2% (95% CI: 0.775-0.950, *P* < 0.0001) with the Youden index 0.6346

The integer value closest to the regression coefficient was used as the score value of the influencing factors, with 2 points for pulmonary infection, hypoalbuminemia, and hyponatremia, and 0 points for uncomplicated pulmonary infection, normal blood albumin level and normal blood sodium level (and thus a total score value of 6). We established a P-Pneumonia, A-Hypoalbuminemia, N-Hyponatremia (PAN) scoring system for predicting early GBS prognosis. The ROC curve that was plotted according to this prediction model (Fig. 1), yielded an AUC of 82.2% (95% CI: 0.775 ~ 0.950, *P* < 0.0001), indicating the validity of this scoring system. We used the Youden index to find the cut-off point, determining that the maximum Youden index was 0.6346 and the corresponding critical score value was 2. The sensitivity of this scoring system to predict the early prognosis of GBS was 0.9091, the specificity was 0.7255, the positive predictive value was 41.7%, and the negative predictive value was 97.4% (Tables 4 and 5).

**Table 4:** ROC of the PAN scoring system

Cut-off value	Sensitivity	specificity	Youden index
0	100.00	0	0
2	90.91	72.55	0.6346
4	63.64	96.08	0.5972
6	9.09	98.04	0.0713

*PAN* P(Pneumonia), A (Hypoalbuminemia), N (hyponatremia)

**Table 5:** PAN scoring system for the short-term prognosis of GBS

Item	Score
**Clinical features**	
With pneumonia	2
Without pneumonia	0
**Laboratory tests**	
Serum albumin < 35 g/L	2
Serum albumin ≥ 35 g/L	0
Serum sodium < 135 mmol/L	2
Serum sodium ≥ 135 mmol/L	0

The PAN score ≥ 2 indicates a poor early prognosis

*PAN* P(Pneumonia), A (Hypoalbuminemia), N (hyponatremia), *GBS* Guillain-Barré syndrome

## Discussion

GBS is an autoimmune-mediated peripheral neuropathy. Additionally, although most patients can be cured with plasma exchange (PE) and intravenous immunoglobulin (IVIG), it still confers a certain degree of disability. Therefore, early prognosis analysis based on risk factors at the beginning stages of the disease can contribute to active intervention and positive guidance that can improve disease prognosis.

In this study, we evaluated differences in gender, age at onset, presence of a history of antecedent infection, presence of cranial nerve involvement during the disease development, presence of pulmonary infection, need for mechanical ventilation, presence of hyponatremia, presence of hypoalbuminemia, and peripheral blood neutrophil-to-lymphocyte ratio between patients with GBS scores ≤ 3 and patients with GBS scores > 3 at discharge. A multivariate regression analysis was used to derive independent predictors of poor early prognosis in patients with GBS, and a clinical prediction model was constructed based on those findings.

Our findings suggest that pulmonary infection is closely related to the early prognosis of patients with GBS. Several mechanisms could underlie the development of pulmonary infections in this disease: pulmonary infections may migrate from antecedent upper respiratory tract infections; or, because airway secretions are not easily eliminated in GBS patients with medullary paralysis, aspiration pneumonia can develop. Similarly, a study by Li et al. in 2019 [5] showed that pulmonary infections in GBS patients were associated with disease severity. Pulmonary infection can result in decreased resistance to pathogens and increased immune responses in patients, giving rise to elevated airway secretions, reduced respiratory function, and respiratory failure. These mechanisms may be responsible for patients’ poor prognoses. Therefore, early prevention of aspiration pneumonia and the selection of appropriate antibiotics for infection control may play pivotal roles in improving the prognosis of GBS patients.

Our study also indicated that hypoalbuminemia was an independent predictor of poor early prognosis in GBS patients. Previous studies have confirmed that albumin exerts its neuroprotective properties through antioxidant effects and regulation of intracellular signaling in neuronal or glial cells [6]. In central nervous system autoimmune diseases such as anti-N-methyl-D-aspartate receptor encephalitis [7] and neuromyelitis optica (NMO) [8], patients tend to have significantly lower serum albumin levels than healthy controls. A retrospective study of 174 GBS patients found that serum protein levels in IVIG-treated GBS patients were significantly associated with short-term and long-term prognosis [9]. A retrospective report on 111 patients by Zhang et al. [10] also concluded that hypoproteinemia suggested a poor prognosis. Yao et al. [11] confirmed a notable decrease in serum albumin levels in patients with GBS in the acute phase compared to healthy controls. They hypothesized that albumin might participate in the antioxidant response in the acute phase of GBS, although no obvious correlation with disease severity was observed.

Coexisting hyponatremia seems to be common in GBS patients. Current opinions consider the syndrome of inappropriate secretion of antidiuretic hormone (SIADH) and cerebral salt wasting syndrome (CSWS) to be the main underlying mechanisms of hyponatremia in these patients, and 48% of GBS patients develop SIADH during their disease course [12]. Several studies have exhibited that low blood sodium levels are associated with GBS severity and poor prognosis [12–16]. Remarkably, hyponatremia may be the initial manifestation of the disease [17]. Because of the different mechanisms underlying SIADH and CSWS, correction of hyponatremia requires different therapeutic principles in each disease: fluid restriction is preferred in SIADH, but CSWS requires fluid and sodium supplementation. Thus, it is particularly important to identify the etiology of hyponatremia and take appropriate therapeutic measures in clinical settings [16].

Our study had some limitations: (1) this study is a single-center retrospective study and has a small sample size, with some design bias and lack of external validation. (2) The study population mainly included cranial nerve-involved patients, and further group comparisons between GBS subtypes were not possible. (3) Due to limited clinical data availability, there are other factors that may affect the early prognosis of GBS (such as ganglioside antibodies and neurophysiological findings) that we did not include in our scoring system. At present, our study has only established a preliminary early prognostic scoring system for GBS. We plan to further validate this scoring system and conduct more in-depth, prospective studies with expanded sample sizes and the inclusion of multiple centers in the future.

In conclusion, this study found that pulmonary infection, hypoalbuminemia, and hyponatremia in GBS patients were independent risk factors affecting the early prognosis of GBS and that scores greater than or equal to 2 on our newly-established PAN-scoring system often indicated a poor early prognosis. Different from previous scoring systems, our prognostic prediction model combines clinical features with laboratory findings, which allows for a more comprehensive assessment and enables clinicians to be more alert to poor prognosis in the early stage and to take appropriate intervention and treatment.

## Supplementary Information

**Additional file 1. **

## Data Availability

The datasets used and/or analyzed during the current study are available from the corresponding author on reasonable request.

## Abbreviations

GBS: Guillain-Barré syndrome
NLR: Neutrophil-to-lymphocyte ratio
ROC: Receiver operating characteristic
AUC: Area under the ROC curve
CI: Confidence interval
HRCT: High-resolution computed tomography
CIDP: Chronic inflammatory demyelinating polyradiculoneuropathy
FPG: Fasting plasma glucose
HFGS: Hughes functional grading scale
PE: Plasma exchange
IVIG: Intravenous immunoglobulin
NMO: Neuromyelitis optica
SIADH: Syndrome of inappropriate secretion of antidiuretic hormone
CSWS: Cerebral salt wasting syndrome
